# Supplementary material for: Physical activity engagement in Eldoret, Kenya, during COVID-19 pandemic
Source: PLOS Glob Public Health. 2022 Apr 13;2(4):e0000339. doi: 10.1371/journal.pgph.0000339 (PMC10021677; doi:10.1371/journal.pgph.0000339)
Supplement: Attachment — Submitted filename: Response to Reviewers.docx [file pgph.0000339.s002.docx]

**Response to Reviewers**

1. Comment 1 noted. Thank you
2. Comment 2 noted. Thank you
3. Comment 3 noted. Data will be made publicly available and uploaded together with revised manuscript.
4. Comment 4 noted and the language, typographical and grammatical errors corrected.

**Reviewer 1**

Thank you for the kind comments, we appreciate.

1. Comment 1: A flow chart should be used to show the samples recruitment adopting both inclusion and exclusion criteria at each stage.

Response: Participants recruitment flow chart has now been included

2. Comment 2: I have not found a single line about ‘How physical activities were measured’? What are the domains the authors considered? How they calculated MET value? Need to describe the measurement of physical activity in a separate paragraph.

Response: The PAs were self-reported but we used the WHO-GPAQ generic showcards attached to the questionnaire for reference by participants. This enabled us to place activity level accordingly. Domains considered were any activity or exercise as categorized in the show card that yielded vigorous or moderate intensity exertion. We have now described this in methods section under “data collection”. We thought it fits here and did not consider a separate paragraph for that.

3. Comment 3: The authors mentioned the aim of this study was ‘to determine the prevalence and regimes of exercise amongst adults one year after the first case of COVID-19 was reported in Kenya’. I am confused about the term ‘exercise’. How the authors examined exercise using GPAQ is misleading here? The authors need to clarify precisely the difference between exercise and physical activity and how GPAQ helped them to quantify exercise?

Response: Throughout the manuscript, we have adopted usage of PA as opposed to exercise, and have now described (see comment 2) how this was done.

4. Comment 4: Authors should describe how they developed the data collection tool and what were the variables they considered to achieve the objective of this study?

Response: Development of the tool has now been described under data collection section. The aim of the study has been fine-tuned in line with how data was collected and analysed based on socio-economic zones participants were drawn from and gender as the major variables considered

5. Comment 5: Authors need to describe the data collection procedure in details. Who collected the data? How privacy was maintained?

Response: This has been done. Explanations provided.

6. Comment 6: Why the authors divided the results into lower-income and higher-income zone? Did it align with the study objectives?

Response: Thank you for pointing this out, the study aim has now been revised to align to this division.

7. Comment 7: In Table 3, the authors reported exercise intensity: mild, moderate and vigorous. However, was it work-related or recreational or transport-related? Authors need to clarify this issue precisely.

Response: we have now clarified that all activities that fitted the various categories in WHO GPAQ questionnaire had their intensity reported in data collected as this fitted the criteria for PA.

8. Comment 8. Throughout the manuscript the authors mixed the term ‘exercise’ and ‘physical activity’. Kindly confirm which was actually you measured?

Response: Thank you for pointing out this oversight. We have since adopted use of PA throughout the manuscript.

**Reviewer 2**

1. General comment

Thank you for the kind comments. The document has been rewritten and language errors corrected.

2. Abstract

Suggestion to indicate that I referred to Eldoret, Kenya has been sorted now.

3. Introductions

Previous paragraph 1 has been deleted and benefits of physical activity included in the new paragraph 1. The authors agree to usage of “physical activity” instead of exercises for this paper. We appreciate the insight. English mistakes have been corrected, repetitions removed and singular ideas fitted into various paragraphs. The aim of the study has been aligned with the rest of the paper sections.

4. Methods

We have clarified that analysis was based on the socio-economic zones in line with the stated aim of the manuscript. We have also clarified that odds ratio reporting was done for significant associations at p<0.05.

5. Results

We have introduced a new table 4 that show which variables had significant differences between zones. We have also introduced a new table 5 as suggested for variables from gender based analyses.

We however think maintaining initial tables that have zone-based percentages and then the overall percentage gives a clearer picture of how intra-zone distributions were before giving an overall picture, and, therefore, seek to maintain.

Data on “Patterns of exercises” that showed significance has been included in the new tables showing variables that significantly differed, but we seek to maintain the table with the general information as well

For the results presented in the text without being aggregated in a Table, the new summary tables cater for that.

The variable on white-collar and blue collar job has been dropped to align the data discussed with the study objective

6. Discussion

Yes in our first paragraph we mention low-income individuals are more active than their higher income counterparts as information in the WHO fact sheets. We attach the caption from the referenced fact sheet under sub-heading “LEVELS OF PHYSICAL ACTIVITY GLOBALLY” and quote it directly thus: *“Levels of inactivity are twice as high in high-income countries compared to low-income countries, ... In high-income countries, 26% of men and 35% of women were insufficiently physically active, as compared to 12% of men and 24% of women in low-income countries. Low or decreasing physical activity levels often correspond with a high or rising gross national product.”*

World Health Organization, *https://*[*www.who.int/news-room/fact-sheets/detail/physical- activity*](http://www.who.int/news-room/fact-sheets/detail/physical-%20%20%20activity)*.* 2020.

The last 2 paragraphs of the discussion have been deleted and/or edited to fit as discussions supported by literature.
