# Supplementary material for: Physical activity engagement in Eldoret, Kenya, during COVID-19 pandemic
Source: PLOS Glob Public Health. 2022 Apr 13;2(4):e0000339. doi: 10.1371/journal.pgph.0000339 (PMC10021677; doi:10.1371/journal.pgph.0000339)
Supplement: Attachment — Submitted filename: Response to Reviewers.docx [file pgph.0000339.s003.docx]

**Response to Reviewers**

**Editor comments**

1. Review of references for completeness and correctness

We have gone through the reference list and find that all the references are relevant as we wanted them to be. We are open to any specific advice you may have on this but from our end, we feel this is okay.

**Comments to the authors**

1. Comment 1. Noted. Thank you
2. Comment 2. Noted. Thank you
3. Comment 3. We hope that the related address for comment 6 satisfies reviewer 1 on this.
4. Comment 4. Noted. Thank you
5. Comment 5. Noted. Thank you
6. Comment 6: Review comments to the author
a) Reviewer 1
1. Your ‘introduction’ should describe the evidence regarding PAL among lower and high-income groups to justify your secondary objectives. I have not yet found anything regarding this.

**Response**: Thank you for pointing us to this. We have now included this evidence. We however note that minimal-recent data on this exists from our set-up in Kenya but data from the region and globally advance the argument.

2. You need to make the Flow chart of sample recruitments more standard. Use arrow under each stem of recruitment & beside the arrow mention the technique you adopted. If any participants excluded, show the numbers & reasons of exclusion.

**Response:** This has now been done.

3. Did you adopt binary logistic regression? What were the factors did you adjust? If not, kindly adopt binary logistic regression to report associated factors.

**Response:** Thank you for this suggestion. We had earlier thought to leave that out but we have incorporated it now. We agree it enriches the paper.

4. Did you apply any technique to control recall bias?

**Response:** We have explained that the selection of included questions was carefully done to only include those that had ease of recall but further explained in our limitations that the bias may not have been fully controlled for given the study design adopted.

b) Reviewer 2. Noted. Thank you
7. Comment 7. Noted. Thank you
